# Generative models of cell dynamics: from Neural ODEs to flow matching

**DOI:** 10.1038/s42003-026-09758-w

**Published:** 2026-02-27

**Authors:** Till Richter, Weixu Wang, Alessandro Palma, Fabian J. Theis

**Affiliations:** 1 Helmholtz Munich, Munich, Germany; 2 Technical University of Munich, Munich, Germany

**Keywords:** Machine learning, Computational models

## Abstract

Neural Ordinary Differential Equations (Neural ODEs) have emerged as a prominent framework for modeling complex dynamical systems. Their ability to describe a system’s underlying dynamical law has attracted attention to applications in life sciences. Single-cell data presents challenges due to noise, sparsity, and the inability to explicitly profile single cells across time. However, pioneering works have demonstrated how Neural ODE-based models can overcome these hurdles, aid mechanistic modeling of cellular development, and approximate population dynamics through the lens of Flow Matching. This article studies why Neural ODEs are suited for modeling the dynamic processes in single-cell data and broader computational health fields, from standard time-series parameterizations to generative models based on optimal transport. We first explore the mathematical properties of Neural ODEs and their application to modeling cellular dynamics. Successively, we zoom into how recent innovations in generative modeling enable efficient and expressive cell state transition modeling through the simulation-free Flow Matching approach. Finally, we present challenges in modeling single-cell dynamics that drive ongoing research in single-cell biology. This work shows that Neural ODEs, as a machine learning framework, are appropriate for modeling dynamic processes in cellular data and promises to advance our understanding of the dynamics in cellular systems.

## Abstract (web summary)

Neural ODEs unify continuous dynamics and neural networks to model cellular dynamics in single-cell omics. This review surveys applications from trajectory modling to gene regulatory network inference, highlighting technical innovations from Optimal Transport to Flow Matching.

## Introduction

### Dynamical perspectives in single-cell biology

Single-cell differentiation - the transformation of a progenitor cell into a specialized entity - plays a pivotal role in the development of complex organisms and in maintaining tissue homeostasis^1^. Central to fields like developmental biology, regenerative medicine, and cancer research, a comprehensive understanding of single-cell differentiation opens avenues for disease treatments, tissue regeneration, and therapeutic cell manipulation^2,3^. Differentiation is driven by gene regulatory networks (GRNs), systems of genes and their regulatory elements that govern cellular functions and response to perturbations. Specifically, during differentiation, GRNs control the activation and suppression of genes, directing the cell’s transition from an undifferentiated to a specialized state. This process is often depicted by Waddington’s landscape^4^, a classical metaphor for cell-fate dynamics.

To capture these GRNs, systems biology has often employed dynamical systems theory, typically constrained to low-dimensional models^5^. These models often rely on coarse-graining, representing the system through a few key variables to make the problem tractable. Yet, the inherent complexity and dynamism of GRNs, marked by multilayered regulation, often exhibit non-linear behaviors beyond the scope of low-dimensional networks. More complex models are essential to unravel these dynamical systems^6^.

Advancements in computational tools and single-cell sequencing have enhanced our understanding of cell differentiation regulation through more complex, high-dimensional GRNs in extensive datasets, including diverse molecular modalities^7^. These vast and intricate datasets offer new insights but present modeling challenges: the data is noisy, sparse, and typically captures only a snapshot of the cellular state. Data-driven methods are emerging that often combine traditional differential equations to capture system dynamics^2,8^. Moreover, ordinary differential equation (ODE)-based generative models offer an expressive framework for learning how distributions of cells evolve.

### Temporal versus observational data

Modern single-cell analysis encompasses temporal, spatial, and spatial-temporal perspectives. Ideally, one would track the temporal evolution of individual cells over time. However, the destructive nature of standard RNA sequencing technologies prevents direct time-course measurements for the same cell across multiple time points. Instead, experiments capture snapshots of cell states as *n*-dimensional data points, resulting in disjoint cellular samples. To model dynamics from such data, one approach is to infer the evolving composition of the cell population over experimental time. Alternatively, single-cell dynamics can be reconstructed by assigning discrete time points *t* ∈ {*t*_0_, *t*_1_, . . . , *t*_*T*_} to individual cells. This can be done using pseudotime, which infers a temporal ordering based on gene expression similarities, or RNA velocity, which estimates directional transcriptome changes to predict future cell states^9,10^. It is important to distinguish between true experimental time (when snapshots are taken at known intervals) and pseudotime (inferred ordering for unsynchronized populations).

Originally centered on inferring cellular trajectories through latent differentiation manifolds^11^, single-cell dynamics research has expanded to include modeling gene regulation^12,13^, estimating cellular velocity^2,14^, and mapping differentiation vector fields^8,15^. These approaches are particularly valuable for non-synchronized populations or steady-state systems where explicit time snapshots are unavailable or lack biological meaning. These advancements have significantly improved our ability to predict the cellular fate and gene regulation^8,16,17^. ODEs have become a popular method for capturing cellular dynamics (Table 1).

**Table 1:** Classical ODE approaches in computational biology

Method	Application Area	Key Contribution
**Kinetic Modeling**		
**Dynamo**^8^	Cell-state transitions	Vector field reconstruction
**RNA-ODE**^71^	Trajectory inference	Gene expression dynamics
**scVelo**^2^	RNA velocity inference	Likelihood-based splicing kinetics
**NN-based Dynamic Inference**		
**PRESCIENT**^72^	Cell fate prediction	Intervention simulation
**veloVI**^73^	RNA velocity	Uncertainty modeling
**Regulatory Inference**		
**CausalKinetiX**^74^	Metabolic networks	Causal structure learning
**Cellbox**^75^	GRN inference	Mechanistic cell responses
**D-CODE**^76^	Model discovery	Symbolic regression
**FLeCS**^76^	GRN inference and use	GRN-based transcription dynamics
**RegVelo**^34^	Velocity and GRN	Unified dynamics framework

When analyzing time-resolved single-cell trajectories from disjoint populations, optimal-transport-based methods have been crucial for learning cellular state transitions across experimental time^18^. Recent technical breakthroughs have further leveraged the connection between the continuous formulation of optimal transport and ODE-based generative models like Flow Matching^19,20^, leading to powerful new approaches for modeling the evolution of cellular distributions over time. This review highlights the emerging role of Neural ODEs in computational cellular biology, showcasing their potential as versatile tools for both mechanistic and generative modeling. Hence, pseudotime is best suited for unsynchronized data, while real-time methods require explicit time labels.

### Configuration space and challenges of pseudotime

**Fig. 1:**
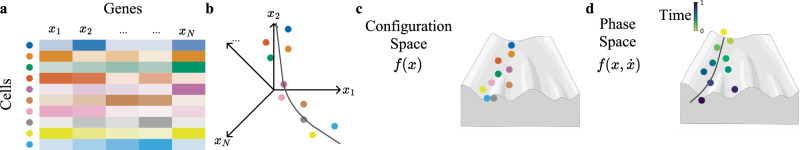
Illustration of Configuration and Phase Space. **a** Cell-by-gene matrix illustrating RNA-sequencing data. **b** High-dimensional data representation. **c** Configuration space in Waddington’s landscape^4^, a metaphor illustrating cell differentiation as a journey from a hilltop to valleys, representing distinct cellular stages. **d** Phase space, extending the landscape to include cellular momentum.

The configuration space^10^ is inspired by classical mechanics and often works in an embedded space via methods like PCA. The trajectory of cell differentiation are captured by uniquely specifying cellular states in the latent space (Fig. 1).

Early research used the configuration space to extract key cellular attributes, such as differentiation stage and pseudotime. Techniques like Monocle^21,22^, Slingshot^23^, TSCAN^24^, Palantir^25^, and Diffusion pseudotime^26,27^ utilize a variety of methods, including ICA, PCA, DDRtree embedding^28^, and diffusion components to predict cellular trajectories. Comprehensive benchmarks comparing these trajectory inference methods on configuration space, such as those conducted in Saelens et al.^29^, provide insights into their relative strengths and limitations across different datasets and biological contexts.

However, pseudotime-based models introduce conflicts when multiple branches exist within the differentiation manifold. If two cells originate from different lineages or branches but are close in pseudotime, the evolutionary process between them is challenging to formulate. With its cell-state-dependent representation, LatentVelo^30^ effectively disentangles the dynamics by conditioning them on this representation. Additionally, some approaches model intrinsic cellular time along processes such as the cell cycle or differentiation by combining pseudotime ordering with dynamic gene regulatory models^31^, though challenges remain in accurately capturing true temporal dynamics from snapshot data.

### Phase space and mechanistic modeling

The study of single-cell dynamics has evolved from the configuration space to include concepts from a phase space that involves a generalized position and momentum (Fig. 1). This space offers a cellular state and transition rates in a unified trajectory. Population Balance Analysis (PBA)^6^ refine the dynamics by modeling potential functions and momenta using a balance equation. RNA velocity^2,32^ determines cell velocity using splicing rate functions to describe mRNA life cycles, while scVelo^2^ uses transient transcription states to describe the entire splicing process. Recent studies such as veloVI^14^ and VeloVAE^33^ focus on kinetic parameter modeling with deep generative models in the RNA velocity framework. However, these methods cannot predict continuous cell-state transitions, marking a gap that newer approaches aim to bridge.

Another approach creates a continuous vector field, mapping position coordinates (e.g., gene expression, principal components) to momentum coordinates. This approach enables differential geometry analysis to measure geometric characteristics like Jacobian, curl, and curvature. Dynamo^8^ utilizes vector-valued support-vector machines for this, revealing gene regulations governing cell fate decisions. DeepVelo^15^ employs a variational autoencoder (VAE) to assess uncertainty in the latent space, quantifying cell instability potentially linked to differentiation priming stages. However, these methods, relying on RNA velocity, are limited by assuming gene independence in velocity estimation, hence not accounting for regulatory effects between genes. In contrast, incorporating Neural-ODEs allows for coupling genes’ dynamics and joint learning of the mapping function and velocity, providing a more accurate representation of cellular dynamics^34^.

Numerous cellular functions, ranging from determining cell fate to responding to perturbations, follow causal systems, such as the GRN. Guided by the principles of Granger causality^35^, stating that causality cannot work against time, dynamical systems allow causal discovery among variables such as genes. Knowing these relationships is crucial for biological systems, as it holds the key to interpreting critical behaviors such as bifurcation, cycling, and other topological phenomena within the differentiation manifold. Distinguishing mere correlations from authentic regulatory interactions within Neural ODEs remains non-trivial. Prior work has employed sparsity assumptions^13,36,37^, *i.e*., that the simplest law is favorable among possible governing laws, but challenges in spurious correlations and unobserved confounders persist. Using interventional data or domain-specific priors could guide the model toward identifying the underlying causal structure^38^, which is crucial to unlocking the true potential of Neural ODEs.

## Theoretical foundations of NeuralODEs and their applications

Motivated by the emergence of data-driven dynamics modeling, this review investigates Neural ODEs—ODEs solved in vector fields that are parameterized by a neural network—as one promising approach. As a potent computational framework, Neural ODEs can naturally model the continuous-time dynamics of cellular states, offering both robustness and explainability.

### Mathematical foundations of Neural ODEs

The rapid rise of neural network (NN)-based methods has transformed how latent spaces are learned from single-cell data. Particularly autoencoders^39^ and VAEs^40^ have been widely adopted to capture meaningful low-dimensional structures, improving batch correction, denoising, and trajectory inference.

Neural Ordinary Differential Equations (Neural ODEs) have emerged as a promising approach for modeling complex system dynamics. Unlike discrete-time Recurrent Neural Networks (RNNs), Neural ODEs excel at capturing the underlying continuous dynamics of noisy and irregularly sampled data^41^. Their causal interpretation allows connections to Structural Causal Models (SCMs) in equilibrium states^35,42^, demonstrated by translating deterministic behavior in ODE systems into a causal framework. With their efficacy in handling noisy data and offering causal insights, Neural ODEs are well-suited for studying biological systems, including single-cell data.

Neural ODEs require the assumption that the data evolves continuously, a process that can be described by a differential equation. We consider a continuous time variable *t* observed at a finite set of discrete time points *t* ∈ [0, *T*]. Specifically, we capture snapshots (e.g., of a cell population ${{{\mathcal{X}}}}$) ranging from ${{{\bf{x}}}}({t}_{0}) \sim {\mathbb{P}}{({{{\mathcal{X}}}})}_{t=0}$ to ${{{\bf{x}}}}({t}_{T}) \sim {\mathbb{P}}{({{{\mathcal{X}}}})}_{t=T}$. In single-cell RNA-sequencing, **x** represents a transcriptome vector with RNA-sequencing counts *x*_*i*_ for each gene *i*.

Neural ODEs model such a dynamical system by learning a NN *f*_*θ*_ ≈ *f* parameterized by *θ* of the true (e.g., first order) ODE

1 $${\dot{{{\bf{x}}}}}(t)=f({{{\bf{x}}}}(t)).$$

By learning the function *f*_*θ*_, a Neural ODE parameterizes a vector field in which the ODE is solved.

**Fig. 2:**
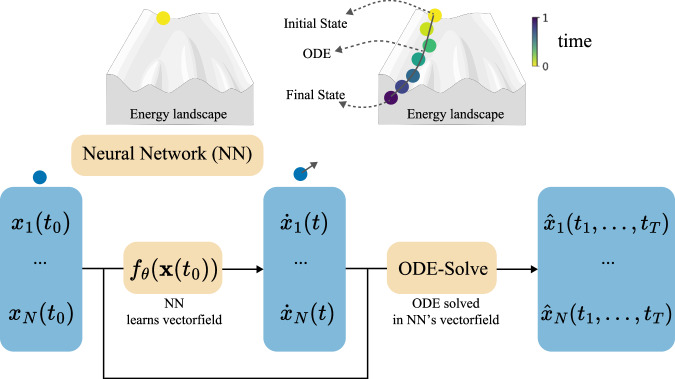
Concept of the Neural ODE framework for single-cell transcriptomics. The Neural ODE takes a cell’s gene expression vector **x**(*t*_0_), ${{{\bf{x}}}}\in {{\mathbb{R}}}^{N}$ at time point *t*_0_ as input. A neural network *f*_*θ*_, parameterized by *θ*, estimates the momentum ${\dot{{{\bf{x}}}}}$, that is together with the initial position the input of an ODE-Solver *ODE-Solve* (in the case of a first-order ODE). Implicitly, the neural network parameterizes the vector field in which the ODE is solved, and the ODE-Solver predicts the expression values at future time points *t*_1_ to *T*.

Figure 2 illustrates the Neural ODE framework. The ODE solver *ODE - Solve*, often sourced from libraries like torchdiffeq^43^, numerically integrates a learned function *f*_*θ*_ to compute a trajectory. Starting from an initial position (i.e., timepoint) **x**(*t*_0_), the solver uses *f*_*θ*_ to predict the momentum ${\dot{{{\bf{x}}}}}$ at that position. It then computes the next position in the trajectory based on this momentum. The solver repeats this process iteratively to generate the entire trajectory **x**(*t*) for *t* ∈ (*t*_0_, *t*_*T*_]:

2 $$\widehat{{{{\bf{x}}}}}\left({t}_{1}\right),\ldots ,\widehat{{{{\bf{x}}}}}\left({t}_{T}\right)=\,{{{\rm{ODE}}}}\; -\; {{{\rm{Solve}}}}\,\left({f}_{\theta },{{{\bf{x}}}}\left({t}_{0}\right),{t}_{1},\ldots ,{t}_{T}\right).$$

The parameters *θ* of the learned function *f*_*θ*_ are updated with a loss function, such as the mean-squared-error (MSE), that evaluates how well the inferred data points $\widehat{{{{\bf{x}}}}}(t)$ match the original data points **x**(*t*) for all *t*.

3 $${{L}}\left({{\widehat{\mathbf{x}}}}(t), {{\mathbf{x}}}(t) \right) 	= {{\mathcal{L}}}\left({{\mathbf{x}}}(t_0) + \int\limits_{t_0}^{t} f_{\theta}({{\mathbf{x}}}(\tau), \tau) d\tau, {{\mathbf{x}}}(t) \right) \\ 	= {{\mathcal{L}}}\left({{\rm{ODE}}}\mbox{-}{{\rm{Solve}}}\left(\, f_{\theta}, {{\mathbf{x}}}(t_0), t_0, t \right), {{\mathbf{x}}}(t) \right)$$

To satisfy applications involving causal systems such as GRNs, we extend the above Neural ODE notation with a notion of causality. We denote the set of causal parents of variable *x*_*i*_ as pa(*i*):

4 $${{\dot{x}}_{i}}(t)={f}_{i}({x}_{{{{\rm{pa}}}}\,{{{\rm{(i)}}}}}(t)),$$

where a causal parent enters into sufficiently smooth function ${f}_{i}:{{{{\mathcal{R}}}}}_{{{{\rm{pa}}}}(i)}\to {{{{\mathcal{R}}}}}_{i}$. The causal interpretation of ODEs is essential for inferring the SCM

5 $${x}_{i}={f}_{i}\left({{{{\bf{x}}}}}_{{{{\rm{pa}}}}{{{\mathcal{M}}}}(i)}\right),\,i\in {{{\mathcal{I}}}}$$

6 $${{{\mathcal{I}}}}:=\{1,\ldots ,N\},$$

where ${{{\mathcal{M}}}}$ is the SCM on the *N* variables ${\{{x}_{i}\}}_{i\in {{{\mathcal{I}}}}}$. The set of causal parents of *x*_*i*_ is given by ${{{{\rm{pa}}}}}_{{{{\mathcal{M}}}}}(i)\subseteq {{{\mathcal{I}}}}\backslash \{i\}$. The SCM is constructed by assigning one node per ODE variable and a directed edge from *x*_*j*_ to *x*_*i*_ if $\dot{{x}_{i}}$ depends on *x*_*j*_.

The Neural ODE framework offers two key advantages. First, the universal approximation theorem ensures that *f*_*θ*_ can model any continuous vector field without prior assumptions about its form. However, in practice, high-dimensional dynamics pose challenges due to limited data and computational constraints. Second, its simple NN foundation enables flexible modifications to address diverse biological questions.

### Applications in single-cell dynamics

**Fig. 3:**
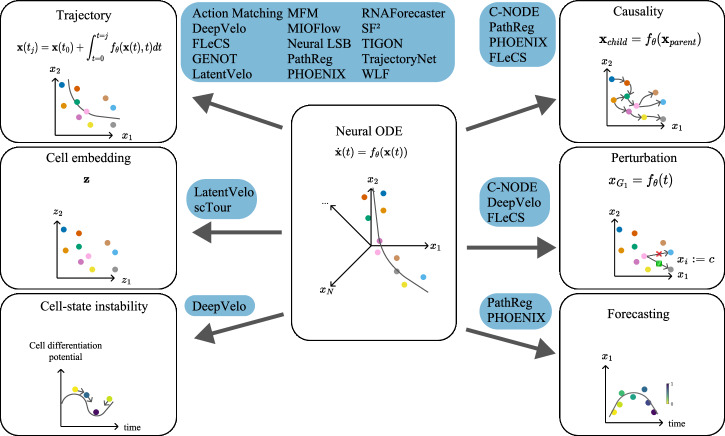
Exemplary applications of Neural ODEs in single-cell genomics. The methods (blue) have included the respective applications but are not restricted to them.

Neural ODEs are particularly valuable in single-cell genomics due to their versatility. As illustrated in Fig. 3 and detailed in Table 2, researchers have adapted the framework for tasks ranging from trajectory inference to the reconstruction of the GRN.

**Table 2:** Methodological overview of Neural ODE approaches in single-cell genomics

Method	Primary Focus	Technical Innovation
**Optimal Transport and Flow Methods**		
**Action Matching**^53^	Irregular sampling	Action-based marginal matching
**CFM**^20^	Continuous trajectories	Flow-based transitions
**MFM**^62^	Complex paths	Metric-based flow matching
**MIOFlow**^54^	Manifold data	Geodesic transport
**Neural LSB**^55^	Stochastic dynamics	Neural SDE framework
[SF]^2^M^59^	Bidirectional analysis	Score-flow matching
**TrajectoryNet**^44^	Population dynamics	CNF with optimal transport
**WLF**^61^	Transport phenomena	Wasserstein optimization
**Network Inference**		
**C-NODE**^37^	Sparse networks	L1 regularization
**PathReg**^13^	Mechanistic analysis	L0-L1 regularization
**PHOENIX**^45^	Large networks	Hill-Langmuir kinetics
**TIGON**^57^	Population dynamics	Unbalanced transport
**Representation Learning**		
**DeepVelo**^15^	Noisy data	VAE uncertainty modeling
**GENOT**^60^	Multi-modal data	Flexible transport
**LatentVelo**^30^	High dimensions	Latent dynamics
**scTour**^47^	Multi-task learning	Joint embedding

#### Trajectory Inference

The concept of trajectory inference is one of the earliest applications of Neural ODEs in single-cell genomics^15,30,44^. Rather than inferring static gene expressions, Neural ODEs predict them as a function of time, thereby providing a trajectory along the phenotypic manifold that contains the cellular differentiation process. Trajectory inference complements pseudotime-based analyses and offers a dynamic view of cellular state transitions.

#### Cell differentiation and mechanistic modeling

The evolution of individual genes along a trajectory, as described by the learned differential equation, promises insights into cell differentiation, e.g., at branching events^13,37,45^, or the concluding cell-state instability^15^. The work in Aliee et al.^37^ highlights the potential of Neural ODEs combined with regularization to uncover system dynamics and causal structures. In Aliee et al.^13^, the focus shifts to the importance of feature sparsity in Neural ODEs for accurately identifying dynamical laws. The study in Hossain et al.^45^ introduces PHOENIX, a framework enhancing the interpretability of ODE representations in gene expression prediction. Finally, DeepVelo^15^ models transcriptome dynamics, emphasizing cell-state instability and developmental driver gene identification.

A further notable innovation stems from the problem of non-identifiability of the governing dynamical system from observational data, shown in Aliee et al.^37^. The assumption of sparse causal connections^36^ inspires the regularization of the input-output connections of the function *f*_*θ*_ to prefer simplicity in the governing system. Such regularization promises sparse relationships in the GRN^13,37^. Building upon these principles, RegVelo^34^ combines RNA velocity and GRN inference into a unified framework, enabling dynamic and interpretable modeling of gene regulation. It achieves superior predictive power for both regulatory interactions and perturbation simulations.

#### GRN inference and causality

Furthermore, GRNs model the (causal) relationship between genes that govern the activation and suppression of genes. The function *f*_*θ*_ gives rise to such a variable interplay that^13,37,45^ investigate for GRN inference. Methods such as RegVelo^34^ can incorporate such a GRN as prior regulation knowledge. Perturbation experiments (gene knockouts, drug treatments) provide crucial causal evidence by revealing which regulatory links change under controlled interventions, helping distinguish causal interactions from correlations. FLeCS^46^ exemplifies this approach by inferring kinetic parameters (regulatory interaction strengths and mRNA half-lives) through coupled ODEs, enabling causal network inference at scale.

#### Representation learning and dimensionality reduction

The embedding analysis is a separate but essential line of investigation in single-cell genomics. Neural ODEs can also operate on data representations transformed by an encoder NN, the weights of which are learned during the process. These embeddings offer valuable perspectives on single-cell data^30,47^.

## Dynamic modeling from time-resolved datasets

**Fig. 4:**
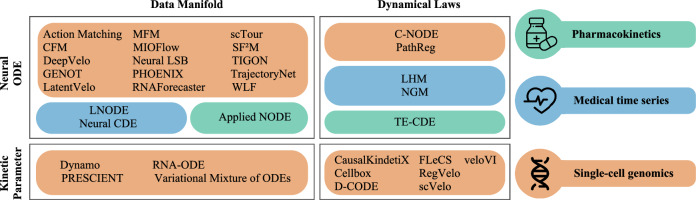
Overview of dynamical models with Neural ODEs (up) and ODEs (down), modeling a data manifold (left) or the governing dynamical laws (right). Applications stem from single-cell genomics and exemplary applications in pharmacokinetics and medical time-series.

Driven by the challenges in learning the single-cell dynamics beyond parametric models and RNA velocity, diverse technical approaches based on cellular manifold estimation and optimal transport emerged, encompassing graph-based single-cell data representations as well as powerful generative approaches (Fig. 4).

### Optimal transport approaches

The scRNA-seq technique destroys a cell during sequencing. Consequently, it is not possible to track the evolving state of an individual cell across experimental time. Instead, time-resolved scRNA-seq involves collecting disjoint snapshots of single cells at successive time points. Cellular evolution can, therefore, be modeled in terms of population dynamics. Specifically, it can be described by a parameterized map that transports distributions of cells forward in time to match future population snapshots. The meaningfulness of the temporal dimension depends on the experimental design: when snapshots are taken at known intervals, the time labels carry real physical meaning as biological time; when they represent an inferred ordering, the temporal axis serves as a modeling construct for population dynamics^26^. Therefore, optimal transport (OT) methods are well suited when discrete time-point data are available as snapshots of cell populations. In pursuing real-time dynamics inference, utilizing discrete OT to deduce the cell state transitions across distinct time points is a promising approach^18,48–50^. In this scenario, Waddington-OT^48^ infers ancestor-descendant fates and modeling regulatory programs, revealing a diverse range of developmental programs from single-cell RNA sequencing profiles. Moslin^49^ couples matching cellular profiles across time points by leveraging both lineage relations and gene expression similarity, Moscot^18^ is a scalable framework for optimal transport in single-cell genomics, enabling efficient reconstruction of developmental trajectories and identification of driver genes across temporal and spatial datasets. JKONet^51^ learns optimal transport via Input Convex Neural Networks by minimizing a parameterized energy function. However, these methods do not yield a continuous vector field but only infer the transport plan across time points^48^, also necessitating an assumption of linearity in velocity between different time points^18,48^. By incorporating a Neural ODE solver and leveraging prior biological knowledge, inferring continuous dynamics guided by vector fields grounded in robust biological hypotheses becomes conceivable^45^.

### Continuous normalizing flows and ODE-based generative models

Inspired by the success of OT in dynamical modeling, several works have explored OT-based regularization techniques for Neural ODEs. These regularizers are incorporated within the continuous normalizing flow (CNF) framework^41^, which models data distributions with a flow of infinitesimal transformations (change of variables formula) from noise to data, using an ODE solver to learn the transformation over time. Infusing OT into CNFs enables training a Neural ODE that matches distributions of samples across time, improving dynamics fitting by minimizing displacement costs while forcing trajectory to reconstruct marginal time points. For example, TrajectoryNet^44^ uses the Kullback-Leibler divergence between real samples and observations integrated over time with a Neural ODE as part of an energy loss to approximate the *L*^2^ Wasserstein distance between single-cell snapshots^52^. Meanwhile,^53^ formulates the optimal vector field matching temporal marginals as the gradient of a function called action, deriving a tractable objective based on kinetic energy minimization. Other methods include the 2-Wasserstein distance regularization of trajectories learned in the latent space of an approximately Euclidean geodesic autoencoders^54^ or extending learning population dynamics to a stochastic setting, representing paths between temporal snapshots as the solutions of Neural SDEs approximating the Schrödinger bridge problem^55,56^. The Schrödinger Bridge problem finds the most likely stochastic process (diffusion) connecting two observed distributions while minimizing entropy relative to a reference process. Finally, OT has been successfully shown to approximate population growth and gene regulatory interactions across the dynamics^57^.

### Flow matching and simulation-free approaches

#### Flow matching

**Fig. 5:**
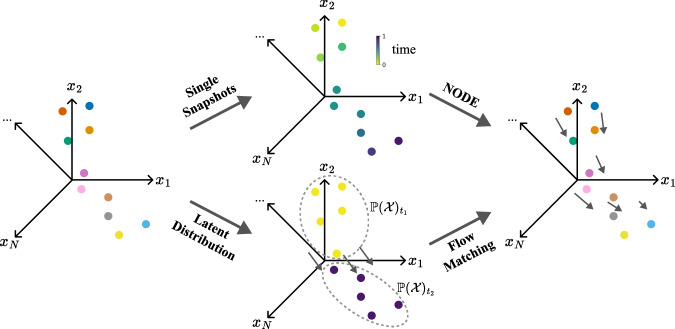
Inferring cellular dynamics through neural ODEs and Flow Matching. Neural ODEs consider snapshots of cell states at discrete (pseudo-) time points *t* ∈ *t*_0_, *t*_1_, …, *t*_*T*_. Flow matching considers latent distributions of cells ${\mathbb{P}}{({{{\mathcal{X}}}})}_{t}$. Both approaches result in a vector field describing cellular dynamics.

Flow matching^19^ simplifies the learning of data transformations by directly minimizing the difference between the learned vector field and the optimal transport vector field, approximated along a straight line connecting samples from consecutive timepoints matched with mini-batch OT (see Fig. 5). Like OT, flow matching is well suited for datasets comprising discrete snapshots of cell populations at successive time points, inferring dynamics without requiring continuous single-cell tracking. This approach avoids expensive ODE integration during training. Besides its generative capabilities^58^, flow matching has also inspired extensions to model single-cell dynamics, such as OT-based regularization to stabilize training and inference for single-cell modeling^20^. Simulation-free score and flow matching ([SF]^2^M)^59^ solves the Schrödinger bridge problem, efficiently modeling high-dimensional cell dynamics without requiring full trajectory simulation. Similarly, generative entropic neural OT (GENOT)^60^ applies flow matching to unbalanced Gromov-Wasserstein problems, extending the framework to handle multimodal single-cell data and developmental trajectories. Wasserstein Lagrangian Flows (WLF)^61^ improve computational efficiency in large-scale cell trajectory modeling by solving transport problems with entropic regularization. Finally, Metric Flow Matching (MFM)^62^ introduces a metric-based flow matching variant, which offers improved geometric fidelity for learning dynamic processes.

### Stochasticity and noise in single-cell dynamics

Single-cell processes exhibit inherent stochasticity due to noisy expression patterns and experimental artefacts. While Neural ODEs model average trajectories, they can be extended to Neural SDEs (Stochastic Differential Equations) to account for random fluctuations and incorporate knowledge on biological stochasticity^63^. Learning stochastic dynamics has been explored in the context of optimal transport through the Schrödinger Bridge problem, which finds the most likely stochastic process connecting two observed cell state distributions, balancing fit and randomness. Established approaches^57,64^ learn to fit stochastic dynamics with neural networks, accounting for cell growth and death in developmental processes. Schrödinger Bridges have been combined with flow models as well in methods like Neural LSB^55^ and [SF]^2^M^59^, capturing stochastic differentiation patterns driven by optimal transport.

## Challenges

Neural ODEs have emerged as a compelling framework for modeling dynamics in single-cell genomics and related computational health disciplines. However, fully addressing the diverse characteristics of biological data faces three predominant challenges from the technical standpoint.

First, understanding the causal relationships and underlying regulatory mechanisms presents a challenge. Guided by the principles of Granger causality^35^, stating that causality cannot work against time, dynamical systems allow causal discovery among variables such as genes. Knowing these relationships is crucial for biological systems, as it holds the key to interpreting critical behaviors such as bifurcation, cycling, and other topological phenomena within the differentiation manifold. Distinguishing mere correlations from authentic regulatory interactions within Neural ODEs remains non-trivial. This challenge is compounded by the difficulty of distinguishing direct regulatory effects from indirect effects mediated by intermediate genes, as well as the presence of feedback loops in GRNs that can confound causal interpretation. Prior work has employed sparsity assumptions^13,36,37^, *i.e*., that the simplest law is favorable among possible governing laws, but challenges in spurious correlations and unobserved confounders persist. Using interventional data or domain-specific priors could guide the model toward identifying the underlying causal structure, which is crucial to unlocking the true potential of Neural ODEs.

Second, the necessity for generalizing beyond interpolation is critical^13^: Models should not overfit the training data but learn the governing laws behind biological dynamics instead. Applications from single-cell genomics, such as driver gene perturbation^65^ and predicting future cell states^15^, require learning the underlying dynamics that govern the evolution of biological systems. Evaluation metrics must extend beyond interpolation, focusing on a model’s ability to robustly capture underlying dynamical laws. This objective is challenging due to, e.g., lacking ground truth and numerous out-of-distribution definitions. Rigorous out-of-distribution testing constitutes a promising research direction.

Third, effectively incorporating real-world data entails accounting for confounding variables ranging from stochasticity to latent influences^6^. To achieve robustness, strategies such as regularization and including biological priors have been proposed^13,45^.

Beyond single-cell genomics, Neural ODEs have demonstrated broad applicability in computational health. As summarized in Table 3, these models support disease monitoring, pharmacology, and biological systems by integrating expert knowledge, capturing stochastic processes, and enabling counterfactual analysis. In genomics, multi-omics techniques provide complementary information across molecular layers^66^, yet integrating these modalities remains challenging^67^. Extending Neural ODEs to further real-world applications thus requires robust strategies for handling heterogeneous data while preserving mechanistic interpretability.

**Table 3:** Neural ODE applications in medicine and pharmacology

Method	Application Area	Key Contribution
**Disease Monitoring**		
**Neural CDE**^77^	Disease detection	Continuous monitoring
**LHM**^78^	Health trajectories	Expert knowledge integration
**LNODE**^79^	Cardiac dynamics	Heart failure progression
**Biological Systems**		
**NGM**^80^	Biological oscillations	Chaotic dynamics
**Neural ODE-PK**^81^	Drug response	Pharmacokinetics modeling
**TE-CDE**^82^	Treatment planning	Counterfactual analysis

## Conclusion and outlook

This article has studied the ODEs framework and its diverse applications in single-cell genomics. We have portrayed single-cell dynamics through the lens of the Waddington landscape, derived the Neural ODE framework in the context of biological application, and reviewed recent literature concerning their technical and practical innovations.

Exploring biological dynamics through computational models holds the potential for illuminating their underlying mechanisms. Despite growing interest in modeling techniques, such as Neural ODEs, the fundamental technical challenges of explainability, generalizability, and robustness persist. Synergistic approaches to improve Neural ODE optimization, such as optimal transport-based regularization, have shown promising results. We anticipate continued innovation by integrating the strengths of diverse modeling paradigms such as optimal transport, classical dynamics modeling, or incorporating regulatory elements from computational biology.

Other advancements in applying Neural ODEs to single-cell data aim to achieve robust learning and causal modeling of the governing dynamical laws. By delving deeper into this domain, we envision revealing the causal mechanisms governing cellular dynamics. Such insights into the underlying dynamical law enable in-silico simulations and predictions, allowing us to anticipate the impact of various factors on biological readouts like single-cell gene expression.

From an application standpoint, Neural ODE methods promise generalizable insights into single-cell dynamics. Their technical versatility allows deployment in numerous downstream applications within and beyond single-cell genomics, including medical time series and pharmacokinetics, highlighting the broad applicability of dynamical systems modeling.

In the broader machine learning landscape, foundation models such as SORA^68,69^ are beginning to capture the dynamic world. While single-cell foundation models largely focus on static settings^70^, the development of such dynamic models offers an intriguing future direction. By advancing Neural ODEs and similar approaches, we come closer to achieving a large-scale model of the dynamic world of cellular processes.

In conclusion, Neural ODEs are an empowering and innovative framework for modeling underlying dynamics in single cells and the broader realm of computational health. The technical innovations of Neural ODEs and their future prospects foreshadow a more holistic understanding of the governing dynamical laws that steer cell differentiation. The presented applications and challenges collectively contribute to a captivating and promising journey, inspiring further advancements in this transformative field.

### Reporting summary

Further information on research design is available in the Nature Portfolio Reporting Summary linked to this article.

## Supplementary information

Reporting Summary

## References

[1] Newman, S. A. Cell differentiation: what have we learned in 50 years? *J. Theor. Biol.* **485**, 110031 (2020). 31568790 10.1016/j.jtbi.2019.110031

[2] Bergen, V., Lange, M., Peidli, S., Wolf, F. A. & Theis, F. J. Generalizing rna velocity to transient cell states through dynamical modeling. *Nat. Biotechnol.* **38**, 1408–1414 (2020). 32747759 10.1038/s41587-020-0591-3

[3] Shi, J., Aihara, K., Li, T. & Chen, L. Energy landscape decomposition for cell differentiation with proliferation effect. *Natl. Sci. Rev.* **9**, nwac116 (2022). 35992240 10.1093/nsr/nwac116 PMC9385468

[4] Waddington, C. H. *The Strategy of the Genes*, 1st edn. 10.4324/9781315765471. Reprinted as eBook in 2014. (Routledge, London, 1957).

[5] Qiu, X., Ding, S. & Shi, T. From understanding the development landscape of the canonical fate-switch pair to constructing a dynamic landscape for two-step neural differentiation. *PLoS ONE* **7**, e49271 (2012). 23300518 10.1371/journal.pone.0049271 PMC3530918

[6] Weinreb, C., Wolock, S., Tusi, B. K., Socolovsky, M. & Klein, A. M. Fundamental limits on dynamic inference from single-cell snapshots. *Proc. Natl. Acad. Sci.* **115**, E2467–E2476 (2018). 29463712 10.1073/pnas.1714723115 PMC5878004

[7] Angerer, P. et al. Single cells make big data: new challenges and opportunities in transcriptomics. *Curr. Opin. Syst. Biol.* **4**, 85–91 (2017).

[8] Qiu, X. et al. Mapping transcriptomic vector fields of single cells. *Cell* **185**, 690–711 (2022). 35108499 10.1016/j.cell.2021.12.045 PMC9332140

[9] Teschendorff, A. E. & Feinberg, A. P. Statistical mechanics meets single-cell biology. *Nat. Rev. Genet.* **22**, 459–476 (2021). 33875884 10.1038/s41576-021-00341-z PMC10152720

[10] Deconinck, L., Cannoodt, R., Saelens, W., Deplancke, B. & Saeys, Y. Recent advances in trajectory inference from single-cell omics data. *Curr. Opin. Syst. Biol.* **27**, 100344 (2021).

[11] Trapnell, C. et al. The dynamics and regulators of cell fate decisions are revealed by pseudotemporal ordering of single cells. *Nat. Biotechnol.* **32**, 381–386 (2014). 24658644 10.1038/nbt.2859 PMC4122333

[12] Qiu, X. et al. Inferring causal gene regulatory networks from coupled single-cell expression dynamics using scribe. *Cell Syst.* **10**, 265–274 (2020). 32135093 10.1016/j.cels.2020.02.003 PMC7223477

[13] Aliee, H. et al. Sparsity in continuous-depth neural networks. *Adv. Neural Inf. Process. Syst.* **35**, 901–914 (2022). Systematically explores the impact of weight and feature sparsity on the generalization and dynamical law identification capabilities of Neural Ordinary Differential Equations (NODEs), introducing a novel regularization method, PathReg, to enhance input-output relation sparsity, and evaluate it across real-world datasets to assess out-of-distribution generalization in dynamical systems.

[14] Gayoso, A. et al. Deep generative modeling of transcriptional dynamics for RNA velocity analysis in single cells. *Nat. Methods* **21**, 50–59 (2024). 10.1038/s41592-023-01994-w PMC10776389 37735568

[15] Chen, Z., King, W. C., Hwang, A., Gerstein, M. & Zhang, J. Deepvelo: single-cell transcriptomic deep velocity field learning with neural ordinary differential equations. *Sci. Adv.* **8**, eabq3745 (2022). 36449617 10.1126/sciadv.abq3745 PMC9710871

[16] Lange, M. et al. Cellrank for directed single-cell fate mapping. *Nat. methods* **19**, 159–170 (2022). 35027767 10.1038/s41592-021-01346-6 PMC8828480

[17] Weiler, P., Lange, M., Klein, M., Pe’er, D. & Theis, F. Unified fate mapping in multiview single-cell data. *Nat. Methods* **21**, 1196–1205 (2024). 10.1038/s41592-024-02303-9 PMC11239496 38871986

[18] Klein, D. et al. Mapping cells through time and space with moscot. *Nature* **638**, 1065–1075 (2025). 10.1038/s41586-024-08453-2 PMC11864987 39843746

[19] Lipman, Y., Chen, R. T. Q., Ben-Hamu, H., Nickel, M. & Le, M. Flow matching for generative modeling. In *The Eleventh International Conference on Learning Representations*. https://openreview.net/forum?id=PqvMRDCJT9t (2023).

[20] Tong, A. et al. Improving and generalizing flow-based generative models with minibatch optimal transport. *Trans. Mach. Learn. Res*. https://openreview.net/forum?id=CD9Snc73AW. Expert Certification (2024).

[21] Trapnell, C., Cacchiarelli, D. & Qiu, X. Monocle: Cell counting, differential expression, and trajectory analysis for single-cell RNA-seq experiments. *Bioconductor* https://www.bioconductor.org/packages/release/bioc/html/monocle.html (2017).

[22] Saunders, L. M. et al. Embryo-scale reverse genetics at single-cell resolution. *Nature* **623**, 782–791 (2023). 10.1038/s41586-023-06720-2 PMC10665197 37968389

[23] Street, K. et al. Slingshot: cell lineage and pseudotime inference for single-cell transcriptomics. *BMC Genom.* **19**, 1–16 (2018). 10.1186/s12864-018-4772-0 PMC6007078 29914354

[24] Ji, Z. & Ji, H. Tscan: Pseudo-time reconstruction and evaluation in single-cell RNA-seq analysis. *Nucleic Acids Res.* **44**, e117–e117 (2016). 27179027 10.1093/nar/gkw430 PMC4994863

[25] Setty, M. et al. Characterization of cell fate probabilities in single-cell data with Palantir. *Nat. Biotechnol.* **37**, 451–460 (2019). 30899105 10.1038/s41587-019-0068-4 PMC7549125

[26] Haghverdi, L., Büttner, M., Wolf, F. A., Buettner, F. & Theis, F. J. Diffusion pseudotime robustly reconstructs lineage branching. *Nat. methods* **13**, 845–848 (2016). 27571553 10.1038/nmeth.3971

[27] Wei, J., Zhou, T., Zhang, X. & Tian, T. Dtflow: inference and visualization of single-cell pseudotime trajectory using diffusion propagation. *Genom. Proteom. Bioinform.* **19**, 306–318 (2021). 10.1016/j.gpb.2020.08.003 PMC8602766 33662626

[28] Mao, Q., Wang, L., Goodison, S. & Sun, Y. Dimensionality reduction via graph structure learning. In *Proc. 21th ACM SIGKDD International Conference on Knowledge Discovery and Data Mining*. 765–774 (Association for Computing Machinery, New York, NY, 2015).

[29] Saelens, W., Cannoodt, R., Todorov, H. & Saeys, Y. A comparison of single-cell trajectory inference methods. *Nat. Biotechnol.* **37**, 547–554 (2019). 30936559 10.1038/s41587-019-0071-9

[30] Farrell, S., Mani, M. & Goyal, S. Inferring single-cell transcriptomic dynamics with structured latent gene expression dynamics. *Cell Rep. Methods* **3**, 100581 (2023). *Available at SSRN 4330809* LatentVelo leverages deep learning to compute low-dimensional gene dynamics, embedding cells in a dynamics-based latent space via a variational autoencoder and modeling differentiation with neural ordinary differential equations, enabling accurate prediction of cell trajectories and superior batch correction by focusing on gene expression dynamics. 10.1016/j.crmeth.2023.100581 PMC10545944 37708894

[31] Ocone, A., Haghverdi, L., Mueller, N. S. & Theis, F. J. Reconstructing gene regulatory dynamics from high-dimensional single-cell snapshot data. *Bioinformatics* **31**, i89–i96 (2015). 26072513 10.1093/bioinformatics/btv257 PMC4765871

[32] La Manno, G. et al. RNA velocity of single cells. *Nature* **560**, 494–498 (2018). 30089906 10.1038/s41586-018-0414-6 PMC6130801

[33] Gu, Y., Blaauw, D. & Welch, J. D. Bayesian inference of RNA velocity from multi-lineage single-cell data. *bioRxiv*10.1101/2022.07.08.499381 (2022).

[34] Wang, W. et al. Regvelo: gene-regulatory-informed dynamics of single cells. *bioRxiv*10.1101/2024.12.11.627935 (2024). 10.1016/j.cell.2026.04.022 42119563

[35] Granger, C. W. Some recent development in a concept of causality. *J. Econ.* **39**, 199–211 (1988).

[36] Schölkopf, B. et al. Toward causal representation learning. *Proc. IEEE* **109**, 612–634 (2021).

[37] Aliee, H., Theis, F. J. & Kilbertus, N. Beyond predictions in neural odes: identification and interventions. *arXiv preprint*10.48550/arXiv.2106.12430 (2021).

[38] Tejada-Lapuerta, A. et al. Causal machine learning for single-cell genomics. *Nat. Genet.* **57**, 797–808 (2025). 10.1038/s41588-025-02124-2 40164735

[39] Eraslan, G., Simon, L. M., Mircea, M., Mueller, N. S. & Theis, F. J. Single-cell RNA-seq denoising using a deep count autoencoder. *Nat. Commun.* **10**, 390 (2019). 30674886 10.1038/s41467-018-07931-2 PMC6344535

[40] Lopez, R., Regier, J., Cole, M. B., Jordan, M. I. & Yosef, N. Deep generative modeling for single-cell transcriptomics. *Nat. methods* **15**, 1053–1058 (2018). 30504886 10.1038/s41592-018-0229-2 PMC6289068

[41] Chen, R. T. Q., Rubanova, Y., Bettencourt, J. & Duvenaud, D. K. Neural Ordinary Differential Equations. In *Advances in Neural Information Processing Systems* Vol. 31 (Curran Associates, Inc., 2018).

[42] Mooij, J. M., Janzing, D. & Schölkopf, B. From ordinary differential equations to structural causal models: the deterministic case. In *Proceedings of the Twenty-Ninth Conference on Uncertainty in Artificial Intelligence.* UAE'13, pp-440–448. 10.48550/arXiv.1304.7920 (AUAI Press, Bellevue, WA, 2013).

[43] Chen, R. T. Q. torchdiffeq. https://github.com/rtqichen/torchdiffeq (2018).

[44] Tong, A., Huang, J., Wolf, G., Van Dijk, D. & Krishnaswamy, S. Trajectorynet: A dynamic optimal transport network for modeling cellular dynamics. In *International conference on machine learning*, 9526–9536 (PMLR, 2020). TrajectoryNet leverages the integration of continuous normalizing flows with dynamic optimal transport to model continuous, non-linear paths in dynamic systems, overcoming the limitations of static optimal transport models by constraining the flow of entities between distributions, showcasing enhanced modeling of cellular dynamics in scRNA-seq data. PMC8320749 34337419

[45] Hossain, I., Fanfani, V., Fischer, J., Quackenbush, J. & Burkholz, R. Biologically informed neuralodes for genome-wide regulatory dynamics. *Genome Biol.* **25**, 127 (2024). PHOENIX advances ODE-based modeling of gene expression dynamics by integrating neural ODEs with Hill-Langmuir kinetics, offering a scalable framework that enhances biological interpretability through sparse representations and prior knowledge incorporation, demonstrated across in silico experiments, yeast cell oscillations, and genome-scale breast cancer studies. 38773638 10.1186/s13059-024-03264-0 PMC11106922

[46] Bertin, P. et al. A scalable gene network model of regulatory dynamics in single cells. *arXiv preprint arXiv*10.48550/arXiv.2503.20027 (2025).

[47] Li, Q. sctour: a deep learning architecture for robust inference and accurate prediction of cellular dynamics. *Genome Biol.* **24**, 149 (2023). 37353848 10.1186/s13059-023-02988-9 PMC10290357

[48] Schiebinger, G. et al. Optimal-transport analysis of single-cell gene expression identifies developmental trajectories in reprogramming. *Cell* **176**, 928–943 (2019). 30712874 10.1016/j.cell.2019.01.006 PMC6402800

[49] Lange, M. et al. Mapping lineage-traced cells across time points with Moslin. *Genome Biol.* **25**, 277 (2024). 39434128 10.1186/s13059-024-03422-4 PMC11492637

[50] Eyring, L. et al. Unbalancedness in neural monge maps improves unpaired domain translation. *The Twelfth International Conference on Learning Representations*. https://openreview.net/forum?id=2UnCj3jeao (2024).

[51] Bunne, C., Papaxanthos, L., Krause, A. & Cuturi, M. Proximal optimal transport modeling of population dynamics. In *International Conference on Artificial Intelligence and Statistics*, 6511–6528 (PMLR, 2022).

[52] Benamou, J.-D. & Brenier, Y. A computational fluid mechanics solution to the monge-kantorovich mass transfer problem. *Numer. Math.* **84**, 375–393 (2000).

[53] Neklyudov, K., Brekelmans, R., Severo, D. & Makhzani, A. Action matching: learning stochastic dynamics from samples. In *International conference on machine learning*, 25858–25889 (PMLR, 2023).

[54] Huguet, G. et al. Manifold interpolating optimal-transport flows for trajectory inference. In *Advances in Neural Information Processing Systems*. Vol. 35, (eds Koyejo, S. et al.) 29705–29718 (Curran Associates, Inc., 2022). MIOFlow introduces a novel integration of dynamic models, manifold learning, and optimal transport through Neural ODEs and a geodesic autoencoder, offering superior interpolation between static population snapshots by embedding data in a geometry-aware latent space, outperforming existing generative models in capturing continuous population dynamics. PMC10312391 37397786

[55] Koshizuka, T. & Sato, I. Neural lagrangian schrödinger bridge: Diffusion modeling for population dynamics. In *The Eleventh* International Conference on Learning Representations (2023). Addresses the challenge of modeling biological population dynamics with coarse temporal data by introducing a method that combines the Lagrangian Schrödinger bridge problem with neural stochastic differential equations, enabling the approximation of both deterministic and stochastic sample trajectories in line with the principle of least action, and demonstrating efficient high-dimensional dynamics estimation.

[56] Bunne, C., Hsieh, Y.-P., Cuturi, M. & Krause, A. The schrödinger bridge between Gaussian measures has a closed form. In *International Conference on Artificial Intelligence and Statistics*, 5802–5833 (PMLR, 2023).

[57] Sha, Y., Qiu, Y., Zhou, P. & Nie, Q. Reconstructing growth and dynamic trajectories from single-cell transcriptomics data. *Nat. Mach. Intell.* **6**, 25–39 (2024). 38274364 10.1038/s42256-023-00763-w PMC10805654

[58] Palma, A. et al. Generating multi-modal and multi-attribute single-cell counts with CFGen. In *The Thirteenth International Conference on Learning Representations.* https://openreview.net/forum?id=3MnMGLctKb (2025).

[59] Tong, A. et al. Simulation-free schrödinger bridges via score and flow matching. In *Proceedings of The 27th International Conference on Artificial Intelligence and Statistics.* https://proceedings.mlr.press/v238/tong24a.html (2023).

[60] Klein, D., Uscidda, T., Theis, F. & Cuturi, M. Generative entropic neural optimal transport to map within and across spaces. Advances. in *Neural Information Processing Systems.* Vol. 37, 103897–103944 https://proceedings.neurips.cc/paper_files/paper/2024/file/bc46e29f91e676747c584ca181cb0ea1-Paper-Conference.pdf (2024).

[61] Neklyudov, K. et al. A computational framework for solving Wasserstein Lagrangian flows. In *Proceedings of the 41st International Conference on Machine Learning*. 10.48550/arXiv.2310.10649 (2023).

[62] Kapusniak, K. et al. Metric flow matching for smooth interpolations on the data manifold. In *The Thirty-eighth Annual Conference on Neural Information Processing Systems*. 10.48550/arXiv.2405.14780 (2024).

[63] Maizels, R. J., Snell, D. M. & Briscoe, J. Reconstructing developmental trajectories using latent dynamical systems and time-resolved transcriptomics. *Cell Syst.* **15**, 411–424 (2024). 38754365 10.1016/j.cels.2024.04.004

[64] Zhang, Z., Wang, Z., Sun, Y., Li, T. & Zhou, P. Modeling cell dynamics and interactions with unbalanced mean field Schrödinger bridge. In *The Thirty-ninth Annual Conference on Neural Information Processing Systems*. 10.48550/arXiv.2505.11197 (2025).

[65] Krumsiek, J., Marr, C., Schroeder, T. & Theis, F. J. Hierarchical differentiation of myeloid progenitors is encoded in the transcription factor network. *PloS one* **6**, e22649 (2011). 21853041 10.1371/journal.pone.0022649 PMC3154193

[66] Trimbour, R., Deutschmann, I. M. & Cantini, L. Molecular mechanisms reconstruction from single-cell multi-omics data with hummus. *Bioinformatics* **40**, btae143 (2024). 10.1093/bioinformatics/btae143 PMC11065476 38460192

[67] Bergen, V., Soldatov, R. A., Kharchenko, P. V. & Theis, F. J. RNA velocity-current challenges and future perspectives. *Mol. Syst. Biol.* **17**, e10282 (2021). 34435732 10.15252/msb.202110282 PMC8388041

[68] Brooks, T. et al. Video generation models as world simulators. 2024. https://openai.com/research/video-generation-models-as-world-simulators **3** (2024).

[69] Zhu, Z. et al. Is Sora a world simulator? A comprehensive survey on general world models and beyond. *arXiv preprint*10.48550/arXiv.2405.03520 (2024).

[70] Szałata, A. et al. Transformers in single-cell omics: a review and new perspectives. *Nat. Methods* **21**, 1430–1443 (2024). 39122952 10.1038/s41592-024-02353-z

[71] Liu, R., Pisco, A. O., Braun, E., Linnarsson, S. & Zou, J. Dynamical systems model of RNA velocity improves inference of single-cell trajectory, pseudo-time and gene regulation. *J. Mol. Biol.* **434**, 167606 (2022). 35489382 10.1016/j.jmb.2022.167606

[72] Yeo, G. H. T., Saksena, S. D. & Gifford, D. K. Generative modeling of single-cell time series with prescient enables prediction of cell trajectories with interventions. *Nat. Commun.* **12**, 3222 (2021). 34050150 10.1038/s41467-021-23518-w PMC8163769

[73] Gayoso, A. et al. Deep generative modeling of transcriptional dynamics for RNA velocity analysis in single cells. *Nat. Methods* **21**, 50–59 (2022). 10.1038/s41592-023-01994-w PMC10776389 37735568

[74] Pfister, N., Bauer, S. & Peters, J. Learning stable and predictive structures in kinetic systems. *Proc. Natl. Acad. Sci.* **116**, 25405–25411 (2019). 31776252 10.1073/pnas.1905688116 PMC6925987

[75] Yuan, B. et al. Cellbox: interpretable machine learning for perturbation biology with application to the design of cancer combination therapy. *Cell Syst.* **12**, 128–140 (2021). 33373583 10.1016/j.cels.2020.11.013

[76] Qian, Z., Kacprzyk, K. & van der Schaar, M. D-code: Discovering closed-form odes from observed trajectories. In *International Conference on Learning Representations* (ICLR, 2022).

[77] Kidger, P., Morrill, J., Foster, J. & Lyons, T. Neural controlled differential equations for irregular time series. In *Advances in Neural Information Processing Systems.* Vol. 33 (eds Larochelle, H., Ranzato, M., Hadsell, R., Balcan, M. & Lin, H.) 6696–6707 (Neural Information Processing Systems Foundation, Inc. NeurIPS, 2020).

[78] Qian, Z., Zame, W., Fleuren, L., Elbers, P. & van der Schaar, M. Integrating expert odes into neural odes: pharmacology and disease progression. *Adv. Neural Inf. Process. Syst.* **34**, 11364–11383 (2021).

[79] Salvador, M. et al. Real-time whole-heart electromechanical simulations using latent neural ordinary differential equations. *npj Digit. Med*. **7**, 90 (2024). 10.1038/s41746-024-01084-x PMC11009296 38605089

[80] Bellot, A., Branson, K. & van der Schaar, M. Neural graphical modelling in continuous-time: consistency guarantees and algorithms. In *International Conference on Learning Representations* (ICLR, 2022).

[81] Lu, J., Deng, K., Zhang, X., Liu, G. & Guan, Y. Neural-ode for pharmacokinetics modeling and its advantage to alternative machine learning models in predicting new dosing regimens. *iScience* **24**, 102804 (2021). 34308294 10.1016/j.isci.2021.102804 PMC8283337

[82] Seedat, N., Imrie, F., Bellot, A., Qian, Z. & van der Schaar, M. Continuous-time modeling of counterfactual outcomes using neural controlled differential equations. In *Proc. 39th International Conference on Machine Learning*. Vol. 162, 19497–19521 (PMLR, 2022).

